# Gamma Delta (*γδ*) T Cells and Their Involvement in Behçet's Disease

**DOI:** 10.1155/2015/705831

**Published:** 2015-10-11

**Authors:** Md. Samiul Hasan, Lesley Ann Bergmeier, Harry Petrushkin, Farida Fortune

**Affiliations:** Centre for Clinical and Diagnostic Oral Sciences, Institute of Dentistry, Queen Mary University of London, Blizard Building, 4 Newark Street, London E1 2AT, UK

## Abstract

Behçet's disease (BD) is a multisystem inflammatory disorder characterized by orogenital ulcerations, ocular manifestations, arthritis, and vasculitis. The disease follows a relapsing-remitting course and its pathogenesis is unknown. Genetic predisposition and immune-dysregulation involving gamma delta (*γδ*) T cells are reported to have a role. *γδ* T cells are atypical T cells, which represent a small proportion of total lymphocytes. They have features of both innate and adaptive immunity and express characteristics of conventional T cells, natural killer cells, and myeloid antigen presenting cells. These unconventional T cells are found in the inflammatory BD lesions and have been suggested to be responsible for inducing and/or maintaining the proinflammatory environment characteristic of the disease. Over the last 20 years there has been much interest in the role of *γδ* T cells in BD. We review the literature and discuss the roles that *γδ* T cells may play in BD pathogenesis.

## 1. Behçet's Disease

Behçet's disease (BD) is a multisystem inflammatory disorder characterised by relapsing episodes of orogenital ulceration, ocular inflammation, and skin and joint lesions in association with other manifestations including vascular, gastrointestinal, and neurological involvement [1, 2]. BD occurs most frequently across the ancient trading (silk) route stretching between the Mediterranean, Middle East, and far East-Asia [2, 3]. The diagnosis is clinically supported by International Study Group for Behçet's Disease (ICBD) criteria, 1990 [4], and recently revised 2014 criteria [5]. Treatment is based on a combination of topical and systemic immunomodulatory agents [6], but they are by no means a cure.

### 1.1. Pathogenesis of Behçet's Disease

Aetiology of BD is thought to be a combination of several factors. The current consensus suggests that the pathogenesis may be triggered by an environmental agent in a genetically susceptible host [7, 8]. There is a strong association between HLA-B*∗*51 and BD suggesting a genetic predisposition. Recent GWAS studies indicated new susceptibility loci for BD, namely, CCR1-CCR3, STAT4, KLRK1-KLRC4, and ERAP1 [9]. Early hypotheses suggested a trigger mechanism focusing on infectious aetiology with bacterial/viral infections and molecular mimicry via heat shock proteins (see below) [10, 11], while current studies focus on immunodysregulation. Here we have reviewed the role of gamma delta T cells in BD.

## 2. Gamma Delta T Cells

Gamma delta (*γδ*) T cells are a minor population (~0.5–5% of total blood) of T cells expressing TCR *γ* and *δ* chain. These cells play a significant contribution to overall T cell function [12]. They have roles in the first line of defence against several microbial infections including malaria and Tuberculosis (TB), immune-surveillance of cancer, and immunoregulation. The *γδ* T cell functions which may be relevant to the pathogenesis of BD are their ability to recognise qualitatively distinct antigens, to protect different sites of body, and their ability to mediate and modulate responses to specific pathogens. This functional diversity and plasticity make them important in diseases including Behçet's where different bodily compartments are affected.

### 2.1. Unique Characteristics of Gamma Delta T Cells

Human *γδ* T cells are generally divided into V*δ*1 and V*δ*2 subset. V*δ*1 is the predominant tissue resident cells whereas V*δ*2 is the major subset in peripheral blood [13] which are not found in mice [14]. These subsets are almost exclusively coexpressed with TCR V*γ*9 chain and commonly called V*γ*9V*δ*2. They are unique in their recognition of low-molecular-weight nonpeptide phosphoantigens, for example, (E)-4-hydroxy-3-methyl-but-2-enyl pyrophosphate (HMB-PP), an intermediate metabolite of mevalonate pathway, and expand rapidly in response to a wide variety of pathogens. The intermediate isopentenyl pyrophosphate (IPP) also selectively activates these cells. Unlike conventional *αβ* T cells, these atypical prototypes have demonstrated characteristics of T cells, natural killer (NK) cells, and myeloid antigen presenting cells.* In vitro *studies have suggested that phosphoantigen activated V*δ*2 T cells expressed a repertoire of antigen presentation and costimulatory molecules including HLA-DR, CD80, CD86, CD40, and CD54. Such antigen presenting phenotypes could in turn prime *αβ* T cells to induce strong adaptive responses [12, 15]. These cells interact with dendritic cells (DCs) to regulate their function and mutually promote each other's maturation. Activated *γδ* T cells can also produce high levels of IFN*γ*, TNF*α*, Granzymes, and IL17 reflecting their role in the effector phase of immune response and also have a regulatory role. Furthermore, pattern recognition receptors (PRRs) such as Toll Like Receptors (TLRs) can enhance their function either directly or via DC activation [16].

Migration properties of *γδ* T cells also differ significantly from *αβ* T cells [17]. More than 80% of V*γ*9V*δ*2 cells are excluded from secondary lymphoid tissues such as lymph nodes (LNs) and Peyer's patches lacking CCR7 which is exclusively expressed by *αβ* T cells. These cells however display inflammatory migration profile instead and this is a characteristic shared by granulocytes, monocytes, immature DCs, and NK cells. Above all, they are highly efficient in providing help for B cells for antibody production including IgM, IgG, and IgA [18]. They express costimulatory molecules such as inducible T cell costimulatory molecule (ICOS), CD40, secrete IL-2, IL-4, and IL-10, and thereby have potential roles in autoimmune and chronic inflammatory diseases apart from their anti-infection and antitumour effects. However, their role in BD pathogenesis is still inconclusive.

## 3. Gamma Delta T Cells and Behçet's Disease

### 3.1. Increased Gamma Delta T Cells in Behçet's Disease

The relationship between *γδ* T cells and BD was first documented in early 1990s when a cohort of BD patients were noted to have higher levels of *γδ* T cells in the peripheral blood mononuclear cells (PBMCs) [19, 20]. However, these findings were not solely specific to BD as similar observations were reported in Systemic Lupus Erythematosus (SLE) but were important enough to suggest a potential role of these cells in the disease. Fortune et al. also noted that a significant increase of *γδ* cells was confined to BD patients with inflammatory arthritis but not the ocular and mucocutaneous group of patients. Later, it was suggested that per-cell activity of *γδ* T cells rather than total number is an important factor in BD mechanism [21]. An increased percentage of these cells, in an activated state, were found capable of secreting cytokines such as IFN*γ* and TNF*α* and thereby might induce the proinflammatory environment observed in the clinical disease [22].

There are at least eight functional V*γ* genes and V*δ* transcripts; however V*γ*9V*δ*2 are reported to be the main *γδ* subtype in human peripheral blood [23, 24]. Increased frequency of this subset has been found in PBMCs of BD patients [25, 26], whereas in another study, the highest restriction of V*δ*3 usage was found [27]. An increase in V*δ*1 T cells in cerebrospinal fluid of BD patients with active neurological disease has also been demonstrated [28].

V*δ*1 is the second major subset of human *γδ* T cells which are mainly located in the epithelia and interact with cells expressing MHC class I polypeptide-related sequences A and B (MICA and MICB) through natural killer group 2 member D (NKG2D) activating receptors [29]. *γδ* T cells work as part of the innate immune response to invading microorganisms by recognizing these invariant molecules. They are thought to influence the nature of the adaptive immune response by secreting IL-4 or IFN*γ*, thus regulating the preferential emergence of Th2 and Th1 CD4^+^ T cells, respectively. In addition, they secrete growth factors essential in maintaining mucosal homeostasis. In this regard, a surprising high frequency of V*δ*1 in the peripheral blood as well as in the mucosal disease group has been observed [23]. Presence of all three V*δ* chains within BD oral lesions indicates that usage of V*δ* chains may vary amongst BD patients and is suggestive of a polyclonal activation rather than oligoclonal one, which further suggests that these unique cells might be responding to a wide variety of antigenic and/or nonantigenic stimuli in BD. In addition, the variability of *γδ* subset distribution may support the notion that different subsets of *γδ* T cells might have different roles to play in disease pathogenesis but very little is known to date [30].

### 3.2. Conflicting Data

There is however conflicting data regarding the presence of this atypical cell population in peripheral blood of BD patients. While some groups [19, 20, 22, 28, 31] reported increased *γδ* T cell number in BD, others [26, 32, 33] presented data with no significant increase. A recent study investigating a relatively higher number of BD patients (*n* = 70) has noted that *γδ* T cells were only slightly increased in the blood with no statistical significance compared to healthy controls [34]. Yamashita et al., 1996, also observed an insignificant increase but it is perplexing that no further explanation is evident in the literature regarding these findings. Similarly, an increase in *γδ* T cell expansion has been observed by some groups in active BD compared to inactive BD [20, 31, 32, 35] but there are reports that have found no differences [26, 31, 34]. On further examination of the proportion of individual subsets such as V*δ*2, similar conflicting data was noted. All these discrepancies might be due to the activation status of the disease, as a reflection of local tissue inflammation compared to peripheral blood *γδ* T cells. Such variation might be dependent on several other factors including disease severity, usage of medications such as immunomodulatory agents, and perhaps other variables, namely, age, gender, ethnicity, and/or environmental factors which have already been found to influence the phenotypic and functional differences of peripheral *γδ* T cells [13].

BD patients are most commonly treated with combinations of various immunomodulatory agents including corticosteroids, azathioprine, methotrexate, mycophenolate mofetil, colchicine, and pentoxifylline and biologics such as tumour necrosis factor alpha inhibitors (TNF*α*-inhibitors; Infliximab) were also found useful [36]. The effect of these immunomodulatory agents including pentoxifylline and Infliximab on *γδ* T cells has been studied in BD patients and studies on pentoxifylline indicated that this medication can inhibit cell expansion, downregulate TNF receptor expression, and also inhibit perforin expression [37]. Infliximab also showed similar effects on *γδ* T cells where it suppressed the production of IFN*γ*, perforin, and Granzyme A (GrA)* in vitro* [36]. Azathioprine was reported to ablate *γδ* T cells (V*δ*2 subtype) in Crohn's disease [38] but very little data is available from BD patients regarding the effect of this agent along with others. However, available data clearly suggest that medications influence *γδ* T cells which may result in the discrepancies observed in BD studies. V*γ*9V*δ*2 subsets can express both activating (NKG2C/D) and inhibitory (CD94/NKG2A complex) MHC class I receptor along with CD16 which has significant functional implications including cellular proliferation and cytokine secretion [39]. In active BD, an increase of the activating receptor NKG2C and CD16 were observed; however another activating receptor, NKG2D, was found decreased [34]. Moreover, *γδ* T cell expansion ratio showed conflicting data as restimulation failed to proliferate these cells which was reported earlier [26]. This suggests that, within BD patients, *γδ* T cells are not a homogeneous population but are of heterogeneous spectrum. This is also supported by the phenotypic analysis of these cells, although very few reports are available, where greater variability was noted [25, 26, 35] and often the study becomes challenging with limited number of cells present.

Patients with BD can develop neurological manifestations [25] and it was found that an increased proportion of *γδ* T cells is not linked to how long these patients have suffered from the disease. Similar findings by Ergun et al., 2001, showed comparable peripheral blood *γδ* T cells count in BD and control groups but significantly increased *γδ* T cells in the skin lesions of patients with BD [33]. It is indeed surprising that active and inactive BD do not always show significantly different proportions of *γδ* T cells suggesting a qualitative rather than quantitative difference which may trigger the *γδ* T cells in BD and those subpopulations may have different roles as suggested by the Yamashita group.

### 3.3. Triggers in Behçet's Disease and Gamma Delta T Cells

*γδ* T cells respond to a wide variety of antigens [40] binding to several nonpeptides. It is possible that *γδ* T cells undergo activation resulting in proliferation in response to the products of microorganisms present in BD patients' oral mucosal ulcers [31]. It has been postulated that, in BD patients, the flora of active oral ulcers, at least in part, drives the expansion of *γδ* T cells. The oral microbiota of these patients is significantly populated by pathogenic* Streptococcus* strains including* S. sanguinis *and* S. mitis* [41, 42]. Interestingly, the expansion induced by oral ulcer microbial products involved V*δ*2 subtype, not the V*δ*1, which supports the findings of increased V*δ*2 cells in BD patients. However, it has been argued that *γδ* T cells in normal healthy individuals also expand in response to bacterial antigens, and thus the trigger for *γδ* T cells in Behçet's to initiate the disease process remains inconclusive. However, other ligands for *γδ* TCR such as heat shock proteins (HSP) could possibly be a trigger for initiating the disease process.

HSP are self-determinants expressed on proteins induced by stress. Cross reactivity between oral mucosal and microbial antigens has received considerable attention [43] where microbial heat shock proteins (mHSPs) showing sequence homology with human heat shock proteins (hHSPs) suggest that they may act as a trigger for inducing proinflammatory cytokine profile, characteristic of the disease [7]. Patients with BD respond to four HSP peptides including HSP65 related to* S. sanguinis* reactively present in BD patients sera and mucosal ulcers underpin a role for HSP in BD pathogenesis and a candidate ligand for *γδ* T cells [16, 44]. Moreover, Stanford et al. demonstrated that an induction of tolerance against HSP was capable of ameliorating BD [41]. A BD specific peptide, p336–351, present within the hHSP60 initiated uveitis in rats and following tolerization, both animal model and human trial showed decreased expression of CCR5, CXCR3, CCR7, and costimulatory molecules including CD28 and CD40 by Th1 cells with little or no IFN*γ* and TNF*α* production and thereby preventing the initiation of BD uveitis. However, in contrast to these data, V*δ*2 cells recovered from intraocular fluid of BD uveitis patients failed to demonstrate HSP65 reactivity but responded to nonpeptide antigens, IPP, which are released by damaged cells following infections including* Herpes simplex virus* (HSV) [32]. This again underlines the greater diversity of these cells in antigen recognition. Above all, compared to healthy individuals, BD cells responded to a significantly greater extent indicating previously primed cell population. Bank et al. also postulated that* in vivo* a second encounter with bacterial products or cross reactive autoantigens may lead to inappropriate activation of *γδ* T cells, which after previous activation may have subsequently migrated in the PBMCs or lymphatic system [31]. Conversely V*δ*2 cells in the periphery may have migrated to the mucosa in response to an antigenic exposure in the mouth and then priming other inflammatory cells at distant sites. This phenomenon is evident in Crohn's patients [45] giving rise to the question of whether BD is an* inside out or outside in* phenomena.

BD can be exacerbated following dental treatment and tonsillitis, suggesting abnormal mucosal immunity in these patients [25]. The distribution of *γδ* T cells suggests that they play a pivotal role in mucosal immunity and thereby a major part in the first line of host defence [46]. This coincides well with that of the organ involvement of BD since ~90% of these patients firstly present oral ulceration which may precede the onset of other symptoms by many years [2]. V*γ*9V*δ*2 is the most studied subset of *γδ* T cells that readily respond to infections and are found to be upregulated in patients with active disease. This may explain the clinical observations that BD activity is often triggered by infection. However,* in vivo* activation of V*δ*1 subset was greater than that of the V*δ*2 subset in HLA-B51-positive patients [47]. This finding again indicates that more than one *γδ* T cell subset may be responsible for disease activation suggesting a far more complex pathological mechanism. Indeed, several disease conditions including Rheumatoid Arthritis (RA), inflammatory bowel disease, psoriasis, and airway inflammation demonstrated that different subsets play different roles such as V*γ*4 and V*γ*1 subsets contributed towards these pathologies whereas they suppressed the development of diabetes in NOD mice and Experimental Autoimmune Encephalomyelitis (EAE) [30].

### 3.4. Immunodysregulation, Gamma Delta T Cells, and Behçet's Disease

**Figure 1:**
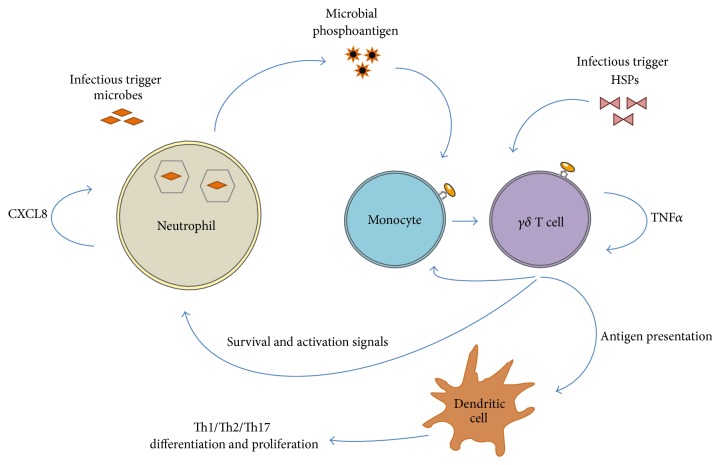
Schematic diagram of the potential interaction of neutrophils, monocytes, and DCs with *γδ* T cells in BD. An infectious trigger (e.g., microbes) results in extravasation of neutrophils and following phagocytosis of the invading microbes, neutrophils release traces of HMB-PP into the microenvironment where *γδ* T cells sense it. Monocytes then might take up or bind this soluble HMB-PP and present it to *γδ* T cells. This interaction triggers TNF*α* secretion, a proinflammatory cytokine along with other similar cytokines including IFN*γ* which promotes *γδ* T cell expansion and drive local chemokine (CXCL8) production that further recruits new neutrophils and monocytes to the site of infection. In addition, activated *γδ* T cells keep providing survival and activation signals to the newly recruited neutrophils and monocytes by secreting TNF*α*. Furthermore, activated *γδ* T cells present antigen to DCs and thus initiate Th1, Th2, and Th17 differentiation and proliferation. Even if the infectious trigger is in the form of a non-HMB-PP source such as HSP60/65, *γδ* T cells can again respond by expanding and keep the interaction active with the neighbouring cells.

*γδ* T cells have been shown to be a strong Th1 and Th17 inducers in experimental models [48] and the percentages of Th17 cells and IL17 have been found to be increased in BD [49]. There is now evidence that the cross talk between lymphocytes and neutrophils might be influenced by the IL17 axis [50]. It was also demonstrated that *γδ* T cells are able to establish effective interaction with neutrophils and monocytes in acute microbial infection responding to bacterial phosphoantigens [51]. Following phagocytosis of pathogenic microbes by neutrophils, *γδ* T cells recognize the bacterial end-product (HMB-PP), establish contact with monocytes, and produce proinflammatory cytokines including TNF*α*. As a result, local *γδ* T cells expand and release chemokines such as CXCL8 (IL8) which then recruits further neutrophils to the site of infection. Activated *γδ* T cells play a pivotal role in this interaction by providing survival and activation signals to newly recruited neutrophils and monocytes. This interaction between these cells may explain the persistent inflammatory symptoms of BD (Figure 1).

In addition, *γδ* T cells express Toll Like Receptors (TLRs 2, 3, 4, 7, 8, and 9) which can prime them to enhance their function [16] and importantly, BD patients have higher TLR expression [52, 53] with TLRs 2 and 4 were upregulated in both monocytes and buccal mucosal cells. In addition, novel splice variants were also expressed which influence the ability of cells to signal the presence of pathogen-associated molecular patterns (PAMPs). It is conceivable that this also occurs in *γδ* T cells and might represent a failure of the negative feedback loop that terminates the inflammatory process. Furthermore, activated TLRs were found on BD neutrophils following exposure to both HSPs and microbial antigens [54]. This indicates the possibility that neutrophils with activated TLRs may provide additional stimulatory signals to *γδ* T cells thus establishing a strong interaction with each other. In addition suppressor of cytokine signalling (SOCS) proteins which negatively regulate the JAK-STAT signalling pathway of cytokine induction appears to be dysregulated in BD [55].

The term “autoinflammatory” disease [56] fits with BD more than “autoimmunity” as there seem to be apparently unprovoked recurrent inflammatory attacks with overexpression of proinflammatory cytokines and no significant autoantibodies. Neutrophils, a key initiator of classical autoinflammation, can go beyond their typical autoinflammatory roles to link the innate immune system with adaptive responses in BD by generating chemotactic signals (e.g., IL8/TNF*α*), expressing costimulatory molecules and releasing proinflammatory cytokines (e.g., IFN*γ*) [57]. Importantly, BD neutrophils were found preactivated [58] and thus might be initiating the intercell cross talk leading to persistent inflammatory response. Moreover, recognition of microbes by *γδ* T cells may require the uptake of whole bacteria by monocytes, neutrophils, or DCs [59]. But the link between these cells with *γδ* in BD has not been studied together in detail. The interplay of different subsets of *γδ* T cells with associated innate and adaptive immune cells during different phases of the disease might be an important clue about the complexity of BD pathogenesis.

## 4. Conclusion

BD seems to be a far more complex disease than often anticipated. An abnormality in the innate immune response along with dysregulated adaptive immunity is likely to be triggering the disease process suggesting a complex interplay of the factors involved. There is evidence to suggest that *γδ* T cells may play a crucial role in this process. The relationship of *γδ* T cells and its surrounding milieu in BD patients may contribute to understanding the pathogenesis of this complex multisystem disease.
